# Quantitative and Ultrasensitive *In-situ* Immunoassay Technology for SARS-CoV-2 Detection in Saliva

**DOI:** 10.21203/rs.3.rs-138025/v1

**Published:** 2021-01-18

**Authors:** Yuchao Chen, Gianluca Roma, Fei Liu, Luke P. Lee

**Affiliations:** 1 WellSIM Biomedical Technologies, Inc., Illumina Accelerator, 200 Lincoln Centre Dr, Foster City, CA, USA; 2 Department of Medicine, Brigham and Women’s Hospital, Harvard Medical School, Boston, MA, USA.; 3 Department of Bioengineering, Department of Electrical Engineering and Computer Science, University of California at Berkeley, Berkeley, CA, USA.

## Abstract

The COVID-19 pandemic has become an immense global health crisis. However, the lack of efficient and sensitive on-site testing methods limits early detection for timely isolation and intervention. Here, we present a Quantitative and Ultrasensitive *in-situ* Immunoassay Technology for SARS-CoV-2 detection in saliva (**QUIT SARS-CoV-2**). Our nanoporous membrane resonator generates a rapid oscillating flow to purify and concentrate SARS-CoV-2 virus in saliva by 40 folds for *in-situ* detection of viral antigens based on chemiluminescent immunoassay within 20 min. This method achieved a detection sensitivity below 100 copies/mL viral load, comparable to the bench-top PCR equipment. The portable QUIT SARS-CoV-2 system, allowing rapid and accurate on-site viral screen with high-throughput sample pooling strategy, can be performed at the primary care settings and substantially improve the detection and prevention of COVID-19.

Currently, the emergence of the novel coronavirus, SARS-CoV-2, is spreading across the world, causing massive healthcare burdens and economic shutdowns at an unprecedented scale.^1,2^ Unfortunately, the daily reported positive cases and deaths keep growing. One of the reasons is the limitations of existing testing capabilities, including a scarcity of convenient tests, time-consuming workflows, and the requirement for samples to be processed in a lab with specialized equipment for sensitive molecular diagnostic methods.

Most current molecular diagnostic technologies rely heavily on PCR, isothermal nucleic acid (NA) amplification, or sequencing-based methods that involve a multi-step approach for sample preparation.^3–7^ While sensitive, the protocol is time-consuming, particularly if viral RNA extraction is required. Furthermore, these medium or high complexity tests require highly skilled laboratory technicians for operation. Another concern is that primers for different genes can be affected by variations in the genomic sequences of the virus due to their rapid mutations, which can generate false-negative results.^8^

Lateral flow assays (LFAs) that probe for the viral antigens or antibody response against the virus are also being actively developed.^9,10^ While allowing timely testing with low cost, these assays lack sensitivity and cannot detect infection at its early stage, when the infected subjects can already transmit virus.^11^ To limit the virus spread, rapid, sensitive, and convenient diagnostic tests are required to identify a person’s infection status as early as possible. A cost-effective technology that permits rapid on-site detection of the virus with high sensitivity and specificity, as well as minimum user intervention (*e.g.,* little hands-on time, low risk) is highly desirable in the on-going pandemic.

Here, we demonstrate a Quantitative and Ultrasensitive *in-situ* Immunoassay Technology for SARS-CoV-2 detection in saliva (**QUIT SARS-CoV-2**). Instead of nasopharyngeal swab specimens, our approach utilizes raw saliva as a specimen (Fig. 1a), which is not only easier for collection but also shows higher detection sensitivity in the first ten days after infection.^12^ The disposable system of QUIT SARS-CoV-2 as shown in Fig. 1b integrates two nanoporous membrane resonators and a detection window into the sample reservoir, allowing rapid virus purification and concentration followed by high-sensitivity analyte detection. Two vibration motors attached to the nanoporous membranes and alternating negative pressure applied on the two outlets together limit the buildup of membrane fouling layers, which would otherwise clog the nanopores.^13^ With this design, the SARS-CoV-2 virus can be isolated from 2 mL of saliva samples and enriched by 40 folds into a final volume of 50 μL within 3 min, inherently enabling a high-throughput sample pooling approach.^14,15^ To minimize hands-on operation and prevent aerosol contamination, a disposable reagent cartridge and two disposable waste containers are integrated with the QUIT SARS-CoV-2 system for sequential injection of sample and reagents as well as the collection of liquid waste (Supplementary Fig. 1). A workstation was developed by integrating a fluidic module and detection unit for automatic system operation and actuation (Supplementary Fig. 2).

The workflow for virus enrichment and detection is shown in Fig. 1c–f. After loading 2 mL of raw saliva from the saliva collector into the reagent cartridge (Supplementary Fig. 3), the sample, mixed with 3 mL of prefilled diluent buffer, is injected into the isolation chamber of the system by applying a positive pressure onto the reagent cartridge. During this process, the saliva sample was prefiltered via a 600 nm nonporous membrane installed inside the reagent cartridge (Supplementary Fig. 1b) to remove large particles (*e.g.,* cells and debris). Alternating negative pressure is then applied onto the two outlets to remove the molecular contaminants (*e.g.,* proteins and nucleic acids) from the 20 nm nanopores (Fig. 1c). The intact viruses are retained inside the isolation chamber due to their larger size between 50 and 200 nm. The vibration motor-induced streaming inside the chamber helps to resuspend the viruses and other particles into the fluid, which can not only limit membrane clogging but also improve virus recovery. After draining the excess liquid, the sample is eventually enriched into 50 μL. The concentrated analyte is located inside the detection chamber at the bottom of the system as shown in Fig. 1d. A mixture of Spike S1 and S2 antibodies labeled with horseradish peroxidase (HRP), the enzyme for chemiluminescent reaction, is then pumped into the system for bioconjugation with SARS-CoV-2 viruses by targeting their surface antigens (Fig. 1e). The antigen-antibody reaction is incubated at room temperature for 5 min before the washing buffer is injected, and the analyte is washed three times to get rid of the unconjugated antibodies and HRPs. During the incubation, the motors keep vibrating to improve the molecular interactions. Eventually, the chemiluminescent substrate is injected into the system for chemiluminescent reaction under the catalyzation of HRP. The emitted luminescent signal for analysis was then detected by a photomultiplier tube (PMT) through an optical fiber aligned with the detection window of the system (Fig. 1e–f).

To validate the system performance, we first studied its capability of virus purification and enrichment by concentrating pooled saliva samples with different viral loads for reverse transcription-quantitative PCR (RT-qPCR) analysis. We enriched 800 μL of each sample into a final volume of 60 μL, which theoretically has 13.3-fold concentration improvement. Compared to the original saliva samples before enrichment, all the samples processed via QUIT SARS-CoV-2 had even lower Ct values as shown in Fig. 2a, indicating a higher detection sensitivity. Especially for the samples with lower virus concentration, more increase in sensitivity was observed. At the concentration of 100 copies/mL, the averaged Ct value was 32.2 after enrichment, while two out of three original samples had no signal been detected in 45 cycles, showing more than 1000-fold improvement in the detection sensitivity. The RT-qPCR analysis demonstrated that our system could effectively concentrate the SARS-CoV-2 virus and improve the detection limit. In our integrated on-chip isolation and detection assay, 40-fold enrichment could be achieved for higher sensitivity improvement.

The QUIT SARS-CoV-2 system was then used to enrich and analyze 15 blind saliva samples with 5 different viral loads from 0 (negative control) to 2500 copies/mL and compared to RT-qPCR as shown in Fig. 2b. The chemiluminescent reaction emits strong optical signals as soon as the HRP substrate is added and maintains the signal intensities on a stable level for minutes (Fig. 2c). After the signal intensity reached a plateau, the coefficient of variation (CV) was generally below 10% without significant fluctuation (Fig. 2d). Therefore, the QUIT SARS-CoV-2 system collected the luminescent signals on the plateau for 30 s to obtain an average detection signal intensity. As the viral load decreased from 2500 to 0 copy/mL, the signal intensities also gradually decreased. The signals detected from the positive samples even at as low as 39 copies/mL could be distinguished from the negative control, rivaling the sensitivity of the RT-qPCR method (Fig. 2b). Above the concentration of 312 copies/mL, the signal intensities of all replications had no overlap with the negative controls, which could be identified as positive results.

Although detection sensitivity usually draws more attention than specificity, more and more concerns have been raised regarding the false-positive testing results.^16–18^ Even a very small false-positive rate (*e.g.,* <1%) may result in a significant proportion of false-positive results, especially when the prevalence of the virus in the population is low.^19^ One unique feature of our QUIT SARS-CoV-2 is that it only isolates and analyses intact virus particles while getting rid of most viral debris, which otherwise can still be detected from a large proportion of recovered patients.^20^ We prepared two aliquots of viral specimens with one of them lysed before the study. The samples were purified and concentrated by the QUIT SARS-CoV-2 system followed by chemiluminescent and RT-qPCR detection of antigen and RNA, respectively (Fig. 2e). In both analyses, the lysed virus’ signal intensities were significantly lower compared to the signal from the intact virus, indicating that viral debris was substantially removed after purification by QUIT SARS-CoV-2.

An internal control system was developed and implemented by using inactivated Influenza A H1N1 virus and alkaline phosphatase (AP) labeled anti-hemagglutinin (HA) antibody to ensure the detection accuracy. The detection process is shown in Fig. 2f. After sample purification and concentration, AP substrate was first added for detection of the internal control. The sample was then washed once to stop light emission and remove background signal before HRP substrate was added for SARS-CoV-2 virus detection. Only when the signal intensity of internal control was higher than a certain level (*i.e.,* >50 RLU), the test was considered valid.

Eventually, we applied the QUIT SARS-CoV-2 system in the analysis of individual saliva samples from ten COVID-19 patients and three healthy controls (Fig. 2g). All the patient samples had Ct values between 18 to 23 analyzed by RT-qPCR (Supplementary Table 1). Our study found most of the patient samples emitted much stronger luminescent signals (>10 folds) than the healthy controls, even exceeding the PMT’s output saturation level at 560 RLU. Although one patient sample, which may experience degradation after melting overnight, was detected with a lower signal intensity at around 140 RLU, it was still differentiated from the negative samples with RLU below 100. Therefore, the QUIT SARS-CoV-2 system successfully identified all the patient samples in the study.

In summary, we have demonstrated a quantitative and ultrasensitive *in-situ* immunoassay to detect SARS-CoV-2 virus in saliva. Compared to the advanced nucleic acids detection methods^21–23^ and other antigen detection approaches such as LFAs and ELISA^9–11^, our technology offers several unique advantages. First, our system allows us to accomplish 40x sample enrichment and highly sensitive chemiluminescent immunoassay in an isolated chamber, enabling rapid sample-to-answer on-site testing (<20 min) with PCR-level detection sensitivity (as little as 39 copies/mL viral load). Due to its high sensitivity and large sample volume load (*i.e.,* 2 mL raw saliva), a sample pooling strategy (*e.g.,* twenty 100μL saliva samples per pool) can be seamlessly implemented for a high-throughput manner without the need for additional equipment. Second, our method could improve the detection specificity by only isolating and detecting intact virus particles while getting rid of viral debris and other molecular contaminants, which otherwise can potentially cause false-positive testing results. Third, the QUIT SARS-CoV-2 system uses a non-invasive method for sample (saliva specimens) collection and virus detection. This method could not only simplify sample collection and minimize interaction between health care providers and potentially infected individuals, but also has higher detection sensitivity in the early-stage infection.^12,24^ With these features, the QUIT SARS-CoV-2 system is ideal for on-site screening testing (*e.g.,* at airports, schools, communities, and outpatient clinics), facilitating timely identification of suspected virus infection.

## Methods

### QUIT SARS-CoV-2 system

The smart system for SARS-CoV-2 virus isolation and detection was fabricated by assembling two symmetrical structures. Two optically transparent polymethyl methacrylate (PMMA) parts were manufactured via computer numerical control (CNC) milling, with an anodic aluminum oxide (AAO) membrane (20 nm pore size, 25 mm diameter, WHA68096002, Sigma-Aldrich) attached on each part using epoxy adhesive. A vibration motor (1597–1244-ND, Digi-key) was immobilized between the AAO membrane and the PMMA structure. Its electric wires are connected to a 3V DC power supply through an opening on the PMMA structure during operation. The two symmetrical parts were assembled using epoxy adhesive to prevent liquid leakage. The disposable reagent cartridge and waste containers were fabricated via stereolithography and integrated with QUIT SARS-CoV-2 system (Supplementary Fig. 1a). The reagent cartridge was connected to the system inlet for reagent injection, while the two waste containers were connected to the two system outlets for waste collection. The reagent cartridge, with a polycarbonate track etch (PCTE) nanoporous membrane (600 nm, PCT0647100, Sterlitech) sealed at the outlet, had six chambers for storage of different reagents including washing buffer, diluent buffer for the saliva sample, antibodies, AP substrate, HRP substrate and peroxide solution (Supplementary Fig. 1b).

### QUIT SARS-CoV-2 workstation

The internal configuration of the workstation is illustrated in Supplementary Fig. 2b. The instrument enclosure was manufactured via selective laser sintering. A microcontroller (Mega 2560, Arduino) was programmed to actuate the system and collect luminescent signals automatically. A diaphragm pump applied 15 kPa positive pressure to the reagent cartridge through a valve group to sequentially inject sample and reagents into the virus isolation chamber. Another diaphragm pump applied 15 kPa negative pressure to the two openings of the waste containers through a pair of three-way valves to collect liquid waste from the isolation chamber. To enable on-site testing, no liquid was circulated inside the workstation. By controlling the two valves, the negative pressure was periodically applied to one waste container every 15 s with another one exposed to the air pressure to achieve negative pressure oscillation. The optical signals emitted from the chemiluminescent reaction were detected by a PMT (H10721–01, Hamamatsu) via an optical fiber (BF23P, Banner) aligned to the detection window on the system. The PMT converted the optical signals into electrical signals, collected by the microcontroller through a transimpedance amplifier and a voltage divider. It was then recorded by the Arduino IDE software (version 1.8.13) for analysis.

### Reagents for virus enrichment and chemiluminescent immunoassay

For system characterization, inactivated SARS-CoV-2 viruses [NATSARS(COV2)-ERC, ZeptoMetrix] were spiked into pooled saliva (991–05-P-250, Lee Biosolutions) as testing samples. Individual saliva samples from COVID-19 patients and healthy controls were purchased from AMSBIO stored at −20 °C until testing. 2 mL of saliva samples were used for testing in each run unless otherwise noted. A saliva collection and purification system (Pure-SAL, Oasis Diagnostics) was used to transfer the saliva sample to the reagent cartridge (Supplementary Fig. 3). 1x TBS buffer (T0537, TEKnova) containing 0.05% tween-20 (T0710, TEKnova) was used as the washing buffer (25 mL) and diluent buffer (3 mL). 100 μL of inactivated Influenza A H1N1 virus (NATFLUAH1N1-ERCM, ZeptoMetrix) was added into the diluent buffer as an internal control. To prepare the antibody mixture, SARS-CoV-2 Spike S1 antibody (GTX635654, GeneTex) and Spike S2 antibody (GTX632604, GeneTex) as well as Influenza A virus H1N1 HA (Hemagglutinin) antibody (GTX127357, GeneTex) were biotinylated via a biotinylation kit (ab201795, Abcam) as per manufacturer protocol. The S1 and S2 antibodies were labeled with streptavidin conjugated HRP (21130, ThermoFisher Scientific). In contrast, the HA antibody was labeled with streptavidin conjugated AP (434322, ThermoFisher Scientific). 1 μL of each antibody was then mixed and diluted into 100 uL by 1x TBS buffer as an antibody solution. 100 μL HRP substrate (A38554, ThermoFisher Scientific) and 100 μL AP substrate (WP20002, ThermoFisher Scientific) were used for chemiluminescent reaction to detect SARS-CoV-2 virus and internal control.

### Reverse transcription-quantitative polymerase chain reaction

RT-qPCR was performed following a nucleic acid extraction-free approach, in which 50 μL of virus sample was first treated with 6.25 μL proteinase K followed by a heat inactivation step and was then directly used as input. A 2019-nCov CDC EUA Kit (10006770, Integrated DNA Technologies) and a Reliance 1 step multiplex enzyme supermix (12010176, Bio-Rad) were used to prepare PCR cocktail including 1 μL N1 probe, 1 μL N2 probe, 6.25 μL enzyme mix, 1.75 μL nuclease-free water, and 15 μL proteinase K-treated virus sample. The RT-qPCR was conducted on a Real-Time PCR Detection System (CFX384, Bio-Rad) following the protocol: 52 °C for 10 min, 95 °C for 2 min, and 45 cycles of 95 °C for 10 s and 55 °C for 30 s. The Ct value for the samples without detection signal was recorded as 45 for quantitative comparison. To obtain the lysed virus sample, viruses were incubated at 65 °C for 30 min for antigen analysis by QUIT SARS-CoV-2 system and treated with proteinase K for 5 min for nucleic acid analysis by RT-qPCR.

### Statistical analysis

Origin 9.1 software was used for graphical representation and statistical analyses. The error bars in the graphical data represent the means ± standard deviations. Statistical significance was determined using an unpaired Wilcoxon test. A p-value < 0.05 indicated statistical significance.

## Supplementary Material

Supplement

## Figures and Tables

**Figure 1.**
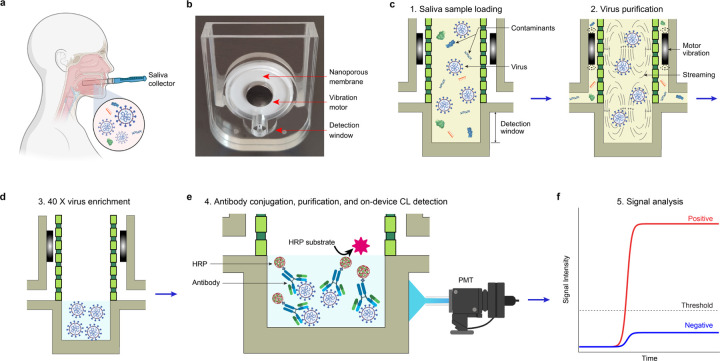
Illustration of the QUIT SARS-CoV-2 system and workflow. (**a**) Illustration showing saliva collection for SARS-CoV-2 virus detection. (**b**) Image of the QUIT SARSCoV-2 system with nanoporous membrane resonators. (**c-f**) Illustration showing the workflow for virus purification, enrichment, and detection using the QUIT SARS-CoV-2 system.

**Figure 2.**
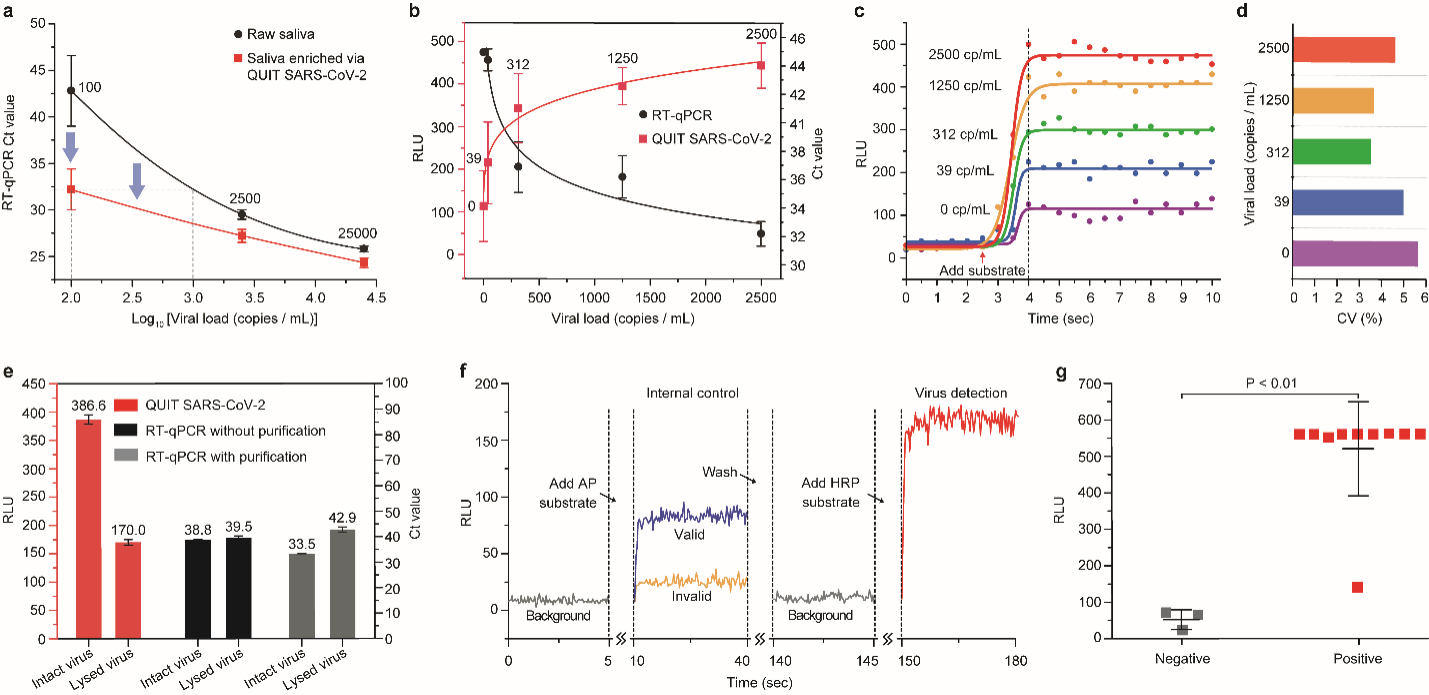
Validation and application of QUIT SARS-CoV-2 system. (**a**) Comparison of RT-qPCR Ct values from samples with and without enrichment by the QUIT SARS-CoV-2 system. SARS-CoV-2 viruses spiked into pooled saliva from healthy donors were used as study samples. (**b**) Characterization of QUIT SARS-CoV-2 using pooled saliva samples spiked with SARS-CoV-2 virus at different concentrations. RT-qPCR was carried out for comparison. Ct value was recorded as 45 when no signal was detected. Pooled saliva without spiked virus (0 copies/mL) was used as negative controls. (**c**) Chemiluminescent signals detected by a PMT at different viral loads. The curves were fitted by the sigmoid function. (**d**) Coefficient of variations (CV) for the signal intensities on the plateau collected in 30 s. (**e**) Comparison of the detection results for intact virus and lysed virus after enrichment by QUIT SARS-CoV-2 system. Chemiluminescent detection by QUIT SARS-CoV-2 and RT-qPCR were used for the characterization of viral antigen and RNA. (**f**) Illustration showing the luminescent signals detected by QUIT SARS-CoV-2 including the detection of internal control and virus. (**g**) Application of QUIT SARS-CoV-2 in the study of COVID-19 patient samples and healthy controls.
