# Supplementary material for: Quantitative and Ultrasensitive In-situ Immunoassay Technology for SARS-CoV-2 Detection in Saliva
Source: Res Sq. 2021 Jan 18:rs.3.rs-138025. Preprint. [Version 1] doi: 10.21203/rs.3.rs-138025/v1 (PMC7814831; doi:10.21203/rs.3.rs-138025/v1)
Supplement: Supplement [file 2be0ef82433d7cb5a032822e.pdf]

Quantitative and Ultrasensitive *In-situ* Immunoassay Technology for SARS-CoV-2 Detection in Saliva

Yuchao Chen[1], Gianluca Roma[1], Fei Liu[1]*, and Luke P. Lee[2,3]*

[1]WellSIM Biomedical Technologies, Inc., Illumina Accelerator, 200 Lincoln Centre Dr, Foster City, CA, USA

[2]Department of Medicine, Brigham and Women's Hospital, Harvard Medical School, Boston, MA, USA.

[3]Department of Bioengineering, Department of Electrical Engineering and Computer Science, University of California at Berkeley, Berkeley, CA, USA.

*Corresponding author: feiliu@wellsimbiotech.com (F. Liu); lplee@bwh.harvard.edu (L. P. Lee)

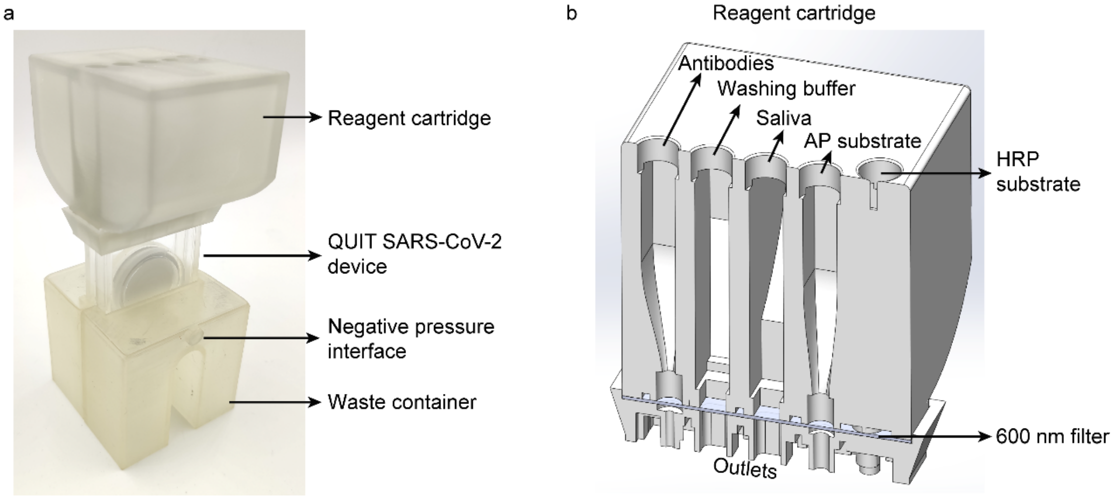

Supplementary Figure 1. (**a**) Image of the disposable components including reagent cartridge, QUIT SARS-CoV-2 system, and waste containers for point-of-care testing. (**b**) Cross-section view of the reagent cartridge design.

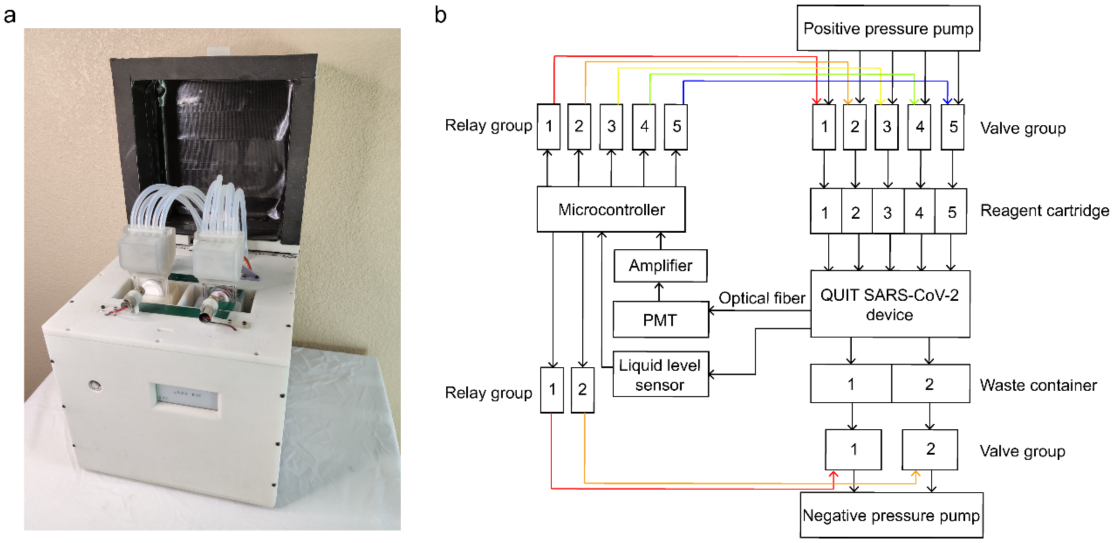

Supplementary Figure 2. (**a**) Image of a prototype of the QUIT SARS-CoV-2 workstation. Each station can run two samples simultaneously. (**b**) Illustration showing the working principle of the QUIT SARS-CoV-2 workstation.

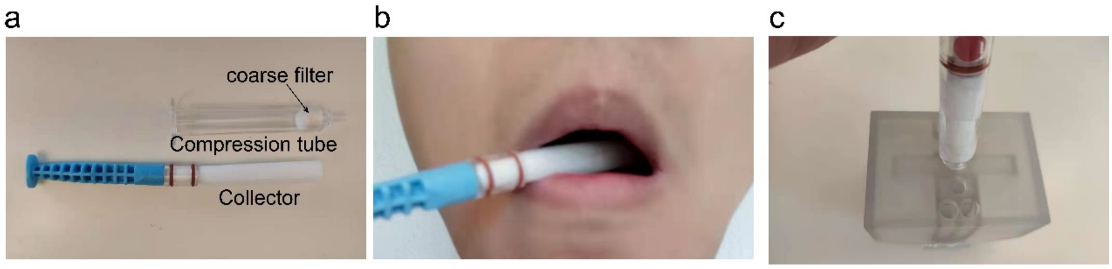

Supplementary Figure 3. (**a**) An image showing the saliva collector including a sponge collector and a compression tube. (**b**) Collect 2 mL of saliva by placing the absorbent sponge into mouth for ~60 s. (**c**) Inject the saliva sample into the reagent cartridge by compressing the absorbent sponge.

Supplementary Table 1. Information of COVID-19 patients and healthy controls in our study.

ID	Age	Gender	Race	Date of test	Ct Value	Test results	Test method
1	75	M	Caucasian	2020/9/10	21.00	+	RT-qPCR
2	58	M	Caucasian	2020/9/9	19.40	+	RT-qPCR
3	57	M	Caucasian	2020/9/10	22.00	+	RT-qPCR
4	40	M	Caucasian	2020/9/11	21.10	+	RT-qPCR
5	48	F	Caucasian	2020/9/10	20.50	+	RT-qPCR
6	50	F	Caucasian	2020/9/10	18.70	+	RT-qPCR
7	62	F	Caucasian	2020/9/12	23.10	+	RT-qPCR
8	63	F	Caucasian	2020/9/10	22.60	+	RT-qPCR
9	24	M	Caucasian	2020/9/9	18.50	+	RT-qPCR
10	51	M	Caucasian	2020/9/10	23.00	+	RT-qPCR
11	50	M	Caucasian	2020/9/10		-	RT-qPCR
12	38	F	Caucasian	2020/9/9		-	RT-qPCR
13	34	M	Caucasian	2020/9/10		-	RT-qPCR
